# Privacy-Preserving Overgrid: Secure Data Collection for the Smart Grid

**DOI:** 10.3390/s20082249

**Published:** 2020-04-16

**Authors:** Daniele Croce, Fabrizio Giuliano, Ilenia Tinnirello, Laura Giarré

**Affiliations:** 1 Department of Engineering, University of Palermo, Viale delle Scienze, ed. 9, 90128 Palermo, Italy; fabrizio.giuliano@unipa.it (F.G.); Ilenia.tinnirello@unipa.it (I.T.); 2 The Department of Computer Science, University of Rome “La Sapienza”, Via Salaria, 113, 00198 Rome, Italy; 3 Department of Engineering, University of Modena e Reggio Emilia, Via P. Vivarelli, 10, 41125 Modena, Italy; laura.giarre@unimore.it

**Keywords:** privacy, secret sharing, distributed, peer to peer, smart grid, overlay networks, P2P, gossiping, overgrid

## Abstract

In this paper, we present a privacy-preserving scheme for Overgrid, a fully distributed peer-to-peer (P2P) architecture designed to automatically control and implement distributed Demand Response (DR) schemes in a community of smart buildings with energy generation and storage capabilities. To monitor the power consumption of the buildings, while respecting the privacy of the users, we extend our previous Overgrid algorithms to provide privacy preserving data aggregation (*PP-Overgrid*). This new technique combines a distributed data aggregation scheme with the Secure Multi-Party Computation paradigm. First, we use the energy profiles of hundreds of buildings, classifying the amount of “flexible” energy consumption, i.e., the quota which could be potentially exploited for DR programs. Second, we consider renewable energy sources and apply the DR scheme to match the flexible consumption with the available energy. Finally, to show the feasibility of our approach, we validate the PP-Overgrid algorithm in simulation for a large network of smart buildings.

## 1. Introduction

Today, Internet of Things (IoT) scenarios where widespread technology is constantly monitoring user activities is crucial to develop algorithms for data collection, monitoring, and control that are guaranteeing the user privacy. All data regarding geolocation, power consumption, visited websites, music, and movie downloads can undoubtedly reveal sensitive information about users’ habits. Exploiting the IoT paradigm, a smart grid is an innovative energy distribution network able to improve the conventional electrical grid so to be more reliable, cooperative, responsive, and economical. The smart grid technology improves two-way communications between utility companies and customers, and exploits near real-time data to make cost-effective and environment-friendly decisions. In the smart grid scenario, traditional and renewable sources are integrated and require an increment of the power system flexibility. Therefore, the control of location-dependent power demand has been tackled with demand response (DR) approaches. DR refers to the possibility of end users to change their consumption patterns in response to a dynamic price signal, or (called load control) to the possibility of directly switching or tuning specific user appliances off during peak demand.

In this paper, we consider DR schemes for a community of smart buildings, aiming at preserving the privacy related to the single user power consumption. The key idea is to extend Overgrid [1,2], a Peer-to-Peer (P2P)-based control scheme for distributed DR, to control a community of users with power consumption comparable to industrial utilities, but without dealing directly with the single residential user (whose contribution in terms of load reduction is limited anyway). Instead, we introduce Privacy-Preserving Overgrid (*PP-Overgrid*) which aims at controlling a group of smart buildings as a whole, protecting the privacy of the users through an innovative Secure Multi-Party Computation mechanism.

We assume that such clusters of buildings are equipped with Internet connectivity and a local communication network to control the electric smart appliances and sensors. We refer to the community of buildings participating to the load control program as an Overgrid. In the present paper, we extend previous results on Overgrid in [1,2] by introducing a mechanism that limits the shared knowledge about user habits to preserve their privacy. The main contributions of the paper are:

- We design PP-Overgrid, a fully distributed P2P architecture able to aggregate and monitor the power consumption of the nodes, while respecting the privacy of the users. The architecture is based on a combination of P2P aggregation algorithm and secure multi-party computation.
- We analyzed the energy profiles of hundreds of real buildings, to distinguish “flexible” energy consumption, useful to be exploited for DR programs.
- Considering real data traces of renewable energy sources, we validate the PP-Overgrid algorithm in simulation for a large network of smart buildings, applying a statistical control of the loads.

Hereafter, we report some Background and Related Work regarding Distribute Load Control, Overlay Networks, and Privacy Preserving schemes. In Section 3, the Overgrid architecture and the P2P protocol are described, together with an aggregation technique. In Section 4, the privacy-preserving generalization is presented and analyzed and finally some experiments and simulations studies are reported in Section 5.

## 2. Background and Related Work

In this section, we review the state-of-the-art solutions for load control and P2P Networks. We also describe some distributed privacy-preserving aggregation schemes, relevant to the understanding of the proposed PP-Overgrid.

### 2.1. Distributed Load Control

Operated at different levels of the grid, load control can alleviate voltage regulation problems (primary level) or matching desired consumption profiles (secondary/tertiary level). Direct Load Control (DLC), for example, is a specific mechanism of demand side management that allows electric utilities to turn specific users’ appliances off during peak demand periods and critical events. Most of the current DLC programs work on thermostatic loads [3,4], such as air conditioners and heating systems because they allow a fine-tuning regulation of power demand.

Multiple load typologies, including interruptible or deferrable loads, have been considered for responding to different frequency components of the regulation signal. For example, in [5], it has been shown that the pool pump control can work at a time scale between the few minutes characterizing the typical control interval of thermostatic loads, and the few hours characterizing the deferral of electric vehicles charging. Internet connections available in most households may enable more flexible programs managed by energy suppliers or third parties [6]. Other solutions are considering the use of enhanced smart plugs which consider user habits and consumption profiles to optimize controllable resources [7].

For a given demand, control signals or control decisions can be programmed by using analytical tools, such as queuing models [8] that have been widely adopted for teletraffic engineering [9]. Indeed, loads on the electrical grid are multiplexed at different aggregation levels (distribution transformers, primary station, price zone), similarly to traffic from data sources multiplexed at a router of a different hierarchical level. In [9], teletraffic analogy has been exploited for sizing the power distribution network as a function of the statistical characterization of the loads, while, in [8], the queuing models are used to study an optimal scheduling of energy suppliers working on renewable sources, without knowing the statistics of the energy production and demand processes. Queuing theory is also used in [10] and [11] for sizing the population of customers subscribing a DLC program under a given maximum tolerable delay for activating the controlled appliances.

### 2.2. Overlay Networks

Overlay networks are particular communication networks built on top of an existing network. With the advent of Internet and peer-to-peer (P2P) networks, overlay networks have attracted much attention for their flexibility and scalability. These networks are a great tool to create application sharing and distribution of large-scale data, multicast application layer services, distribution of On Demand video contents and video streaming, etc. Overlay networks are efficiently used to provide data redundancy, data identification, data search, data persistence guarantees, authentication, and anonymization. They also offer efficient, scalable and robust routing architectures, combined with fault tolerance, load balancing, and explicit geographical knowledge. Since energy systems are intrinsically distributed, the mapping between the energy grid and the network overlay is a straightforward and promising approach.

A particular case of overlay networks are P2P networks, in which the nodes, the so-called peers, all have equivalent functionality. Each pair of nodes can communicate directly or via other peers using routing protocols, and there is no centralized control or hierarchical organization. Generally, P2P networks require no special administrative procedures to be realized and therefore can easily grow both in terms of nodes and data. They are particularly robust to errors and failures and have excellent performance thanks to their decentralized nature.

There are two main classes of P2P overlay networks: structured and unstructured. In structured overlay networks, the network topology is rigidly controlled and the content is stored in specific locations of the network (not on random nodes) so that data search is more efficient. Structured P2P networks usually use distributed hash tables (DHTs) to organize data allocation. Unstructured overlays, instead, are based on looser protocols, where nodes do not have a priori knowledge of the topology, and use flooding mechanisms with limited range to search for content on the network. These mechanisms are highly efficient to locate popular and replicated content but are less efficient in contexts in which the data are sparse. Moreover, the load of each node grows linearly with the number of queries. Despite this, unstructured overlay networks suffer much less overhead than those based on DHTs. As a result, today they are more prevalent on the Internet than the former. For more information on P2P networks, see [12].

A particular use case of overlay networks is the aggregation of distributed data. Data aggregation algorithms determine global properties in the network that can be used in other applications. Examples of aggregate functions are SUM, AVERAGE, MIN, MAX, quantile, etc. In recent years, various algorithms for distributed computation of these functions have been developed, with different advantages and disadvantages in terms of accuracy, robustness, computational complexity, and communication overhead, as discussed in [13]. As we will explain, we use such algorithms in the implementation of Overgrid: in particular, we exploit the Flow Updating [14] algorithm to monitor the average energy consumption of the nodes in the grid. The Flow Updating algorithm is an efficient scheme that has been proven to be fault-tolerant to both message loss and node crashes, and its convergence speed has been studied in [15].

### 2.3. Privacy-Preserving Schemes

Several solutions exist to perform privacy-preserving data mining [16]. Thus far, two main approaches have been proposed: modifying the data before transferring it to the data aggregator (in such a way that the total results are not compromised) or relying on groups of users that must work together to provide aggregate results. Data modifications can lead to mining errors if random noise is used [17] or may become complex when using homomorphic data encryption [18]. Instead, in PP-Overgrid, we employ the second approach based on Multi-Party Computation (MPC) protocols [19]. These algorithms were developed for aggregating information collected from multiple users, without disclosing any information of the single users. Usually, multi-party computing architectures rely on multiple (independent) nodes that deal with randomized versions of the user information provided by the input peers. Several data distribution solutions between nodes exist, based on logical [20] or arithmetic circuits [21] or linear secret sharing [22]. In particular, linear secret sharing mechanisms e.g., Sharemind [23], SEPIA [24], and P4P [25], have been proven to scale well with the increase in the number of users.

PP-Overgrid is based on scalable MPC techniques, exploiting secure functions that can be expressed as iterative data sums. While previous works such as [25] and [26] assume that nodes never leave the network, in PP-Overgrid, the network topology may be unstable during the execution of the data mining operation.

## 3. The Overgrid Concept

The idea in Overgrid is to generalize the concept of a microgrid: the energy resources under control for imposing a desired energy balance with the main grid are not physically localized in a given region, but rather are aggregated regardless of their placement on the energy grid. Buildings joining the Overgrid, also called nodes, are equipped with controllable loads, local generators, and storage systems, which can be jointly exploited for controlling the power demand. Aggregation in an Overgrid can be based on different criteria, such as the type of utilities and the consumption behaviors of the nodes, the level of building exposure to sunlight (which in turns affects the operation of air conditioning systems), the presence of power sources/accumulators, as well as the presence of loads due to electric vehicles. Without loss of generality, in this paper, we will limit the analysis to a single cluster of homogeneous nodes, i.e., a group of smart buildings with different power consumption profiles but uniform capabilities.

### 3.1. Architecture Overview

**Figure 1:**
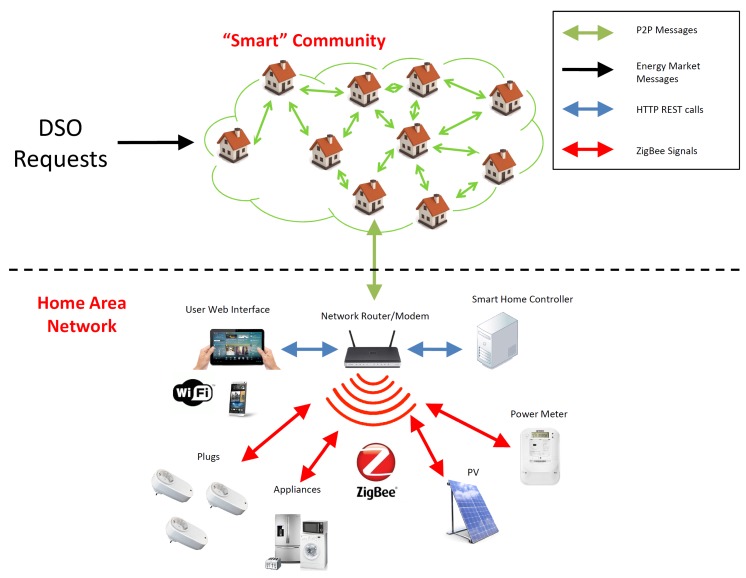
Reference scenario for the Overgrid.

As shown in Figure 1, Overgrid is a *virtual community* of buildings participating in a distributed load control program, by means of an overlay P2P network where each node is a peer in the network. Here, for distributed load control program, we mean the possibility to adapt the aggregated power demand of all the nodes belonging to the Overgrid, in order to satisfy a dynamic power constraint provided by the Distribution System Operator (DSO) without the usage of a central server. Once the logical aggregation is defined and the P2P network is created, the Overgrid is operated as a generic microgrid, collecting the power consumption characteristics of each peer, distributing the DSO requests to modulate the electrical load and implementing the load control (on the individual peers), according to the distributed Demand–Response (DR) paradigm.

Regarding the physical communication network between nodes, we assume that each Overgrid node is equipped with an Internet connection (towards other nodes) as well as a local connection (through WiFi, ZigBee, power line, etc.) for controlling appliances, smart plugs, storage, and production systems. Over this physical connection, the nodes in Overgrid form an unstructured P2P overlay based on Gossip protocols, a commonly used P2P paradigm allowing fault tolerant and scalable information diffusion across the network, totally decentralized and with low network overhead [27]. Gossiping protocols exploit periodic exchange of messages between nodes. The scalability comes from the fact that each node is in communication with a subset of other nodes (typically much smaller than the entire size of the network), which represents the neighbors of the node. Periodically, each node selects another node from its neighbors and exchanges messages containing the local view of some network parameters. Thanks to such a periodic exchange of messages, each node independently carries out a plurality of distributed functions to maintain the P2P overlay structure, distribute information to its neighbors, aggregate data, and operate the DR control, as detailed in [1].

Once a decision on power reduction is taken by a node, a local energy management system is used to map such a decision into a specific control action on the building flexible loads, e.g., tuning of the temperature on the heating/cooling systems, switching off smart appliances, etc., also taking into account the preferences of the users. For example, Figure 1 shows the local energy management system composed of a local server, as well as an interface for defining the load control policies desired by the user by means of a common HTTP protocol. The implementation of the fine-grained local control and the technologies to be used are out of the scope of this paper.

### 3.2. The Distributed Demand Response Scheme

In Overgrid, the nodes monitor a set of local and global parameters needed to implement the distributed DR. In particular, to follow the DSO requests, each node must: (i) receive the DSO request and contribute to spreading it to other nodes; (ii) estimate the total consumption of the network; and (iii) compute a reduction on the current power demand (potentially different for every node) to comply with the DSO request. All of these tasks can be executed by means of the gossiping protocol, which is used for disseminating the information about the DSO request and for implementing a distributed evaluation of the total power demand.

Different approaches can be exploited for estimating the total power demand. We chose to exploit a distributed aggregation technique, called Flow Updating [14], which is very robust for error-prone overlay networks. The scheme is based on the concept of mass saving of the network: starting from the local variable available in each node (the *local* power demand), the nodes iteratively update their estimates on the *total* power demand by averaging the local value with their neighbors estimates. In more detail, Flow Updating uses the concept of *flow* in graph theory and computes the average directly from the input (local) value and the contribution of the flows along graph edges to the neighbors:

(1) $$e_{i}=v_{i}-\sum_{j\in n_{i}}f_{ij},$$

where, at each node *i*, the local estimate $e_{i}$ of the average across the network is computed as the local node value $v_{i}$ minus the sum of the “flows” $f_{ij}$ from node *i* towards each neighbor *j*. The algorithm works as follows: each node *i* stores the flow $f_{ij}$ (initially set to 0) to each neighbor *j*; the node *i* sends a message to node *j* containing information on flow $f_{ij}$ and node *j* updates its own flow $f_{ji}$ with $-f_{ij}$, simply overwriting the previous value. The flows are simply the difference between the local and the neighbor estimates, as defined in [14]. By iterating the estimation process multiple times, each nodes’ estimate converges towards the global average value of the network.

**Figure 2:**
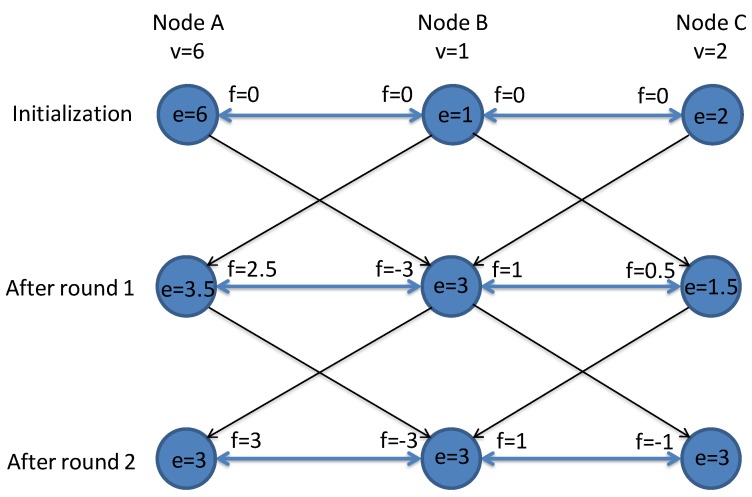
Example of the Flow Updating algorithm with three nodes.

Figure 2 shows an example with three nodes. The local estimate *e* is first initialized with the node’s value *v* (6, 1, and 2, respectively), while flows are set to 0. Then, in the first round, each node sends messages to its neighboring nodes containing the flow towards it and the local estimate (e.g., A will send to B f = 0 and e = 6). At reception of the packets, the nodes update their estimate using Equation (1), i.e., comparing the internal value and the sum of the flows with the neighbors. The results obtained after round 1 are shown in the second row of the figure, where flows “compensate” the difference between estimates: the estimates in the center of the node circle are equal to the flow plus the estimate of the neighbor (at the previous step). The algorithm continues in the same way and converges after the second round. The fast convergence of this technique has been widely studied and demonstrated in [14,15], comparing Flow Updating against other state-of-the-art methods based on Push-Sum Protocol, Push–Pull Gossiping, Distributed Random Grouping and Push-Synopses.

The algorithm allows all nodes to converge to the same result (usually the average of a certain variable, but other functions are possible, e.g., counting and summing), independently of the network topology and dynamically adapting to network changes. In Overgrid, we use Flow Updating to estimate the average power *p* consumed by the nodes and the total number of nodes *N* currently in the network. For this second evaluation, one single node (e.g., the one that starts the Overgrid) is initialized with a local parameter equal to 1 and all the other nodes with a local parameter equal to 0: the number of nodes is given by the reciprocal of the resulting average value. From these two estimates, it is possible to compute the total power demand P=pN consumed by the entire network and, importantly, this parameter is available to each node. This is necessary so that, when a node receives a DSO request, it can modify its power consumption accordingly.

### 3.3. The Load Control Function

Finally, in order to satisfy the DSO request (assumed to arrive every 15 minutes), the combined action of all the nodes in the network is needed. Let $P_{DSO}\leq P$ be the maximum power demand enforced by the DSO into the Overgrid. Each node belonging to the Overgrid has to reduce its power demand in order to meet such a constraint. To achieve this goal, a distributed logic is carefully defined, avoiding all nodes reacting at the same time to the request (causing possible instabilities) and that the requested power reduction is balanced among different peers (avoiding fairness problems). For the first issue, we implemented a random delay mechanism that postpones the power reduction of the nodes for a certain amount of time (*back-off*) to de-synchronize the nodes among them. For the second issue, we assumed that each load can be fine-tuned, in order to implement a power reduction proportional to $P_{DSO}/P$ for each specific load. This approach can be generalized in case of non-tunable loads, by switching off each specific appliance with probability $1-P_{DSO}/P$. In more complex scenarios, other fairness measures could also be employed, for example tacking into account different class of nodes (e.g., industrial vs residential), different priorities (e.g., public safety), or different pricing levels (“premium” users might be less affected compared to “basic” consumers).

## 4. Privacy-Preserving Overgrid

The distributed architecture of Overgrid avoids the presence of a central data-center, which in principle could easily collect consumption data to profile single user behaviors. Indeed, data on users’ power demand can be very sensitive, revealing, for example, the presence (or the absence) of people at home or the number of people living in the same building. However, even using a decentralized P2P scheme like Flow Updating, neighbor nodes exchange information about their real consumption at the first iteration of the scheme for computing the average power demand of the Overgrid, or when a new node joins the Overgrid. Since neighbor nodes might not trust each other, this operation could represent a data breach.

**Figure 3:**
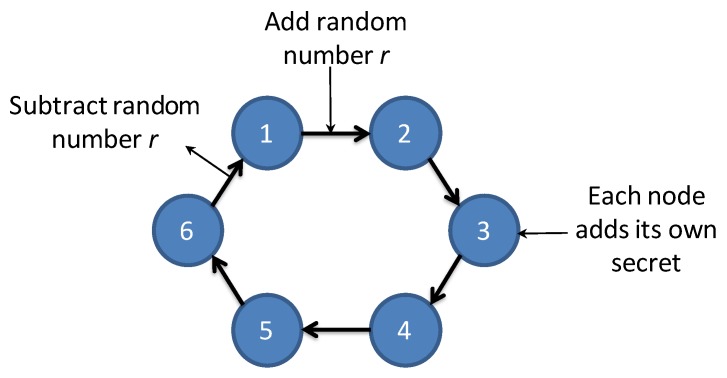
Secure Sum implementation example.

We propose an extension of the distributed DR scheme implemented in Overgrid, in order to mitigate the problem of data breaches between neighboring nodes. The scheme, called Privacy-Preserving Overgrid (PP-Overgrid), combines the original DR algorithm with a Secure Secret Sharing (SSS) scheme [28]. In particular, we use a Secure Sum Computation (SSC), to privately sum the secrets of *n* nodes, using a distributed implementation like the one shown in Figure 3. The algorithm works as follows: assume each node owns its secret $s_{i}$, and that the sum, $\sum_{i}^{n}s_{i}$, lies in the range [0,q). The first node 1 adds a random value *r* to its secret $s_{1}$ modulo *q*, obtaining $r_{1}=\left(r+s_{1}\right)mod\;q$ and sends it to node 2. Then, node 2 adds its own secret to $r_{1}$, obtaining $r_{2}=\left(r_{1}+s_{2}\right)mod\;q$ and sends it to node 3, and so on until the last node *n* sends $r_{n}$ back to node 1. Finally, node 1 subtracts the initial random value *r* and determines the sum $\sum_{i}^{n}s_{i}$.

In PP-Overgrid, the idea is replacing each node with a set of *n* logical sub-nodes, each one with a specific power demand, whose sum corresponds to the real demand of the nodes. For each node *i*, the power demand $sh_{ij}$ of a generic sub-node *j* with j≤n−1 is randomly generated as in the so-called trivial secret sharing scheme, while the last power demand $sh_{in}$ is computed in order to guarantee that the total demand $\sum_{j}^{n}sh_{ij}=p_{i}$ corresponds to the real demand $p_{i}$ of node *i*. The use of logical sub-nodes guarantees that information is not disclosed unless the *n* shares are summed together. If one share is missing, no information can be retrieved (i.e., results become random). This on–off privacy property descends from the homomorphic property of the SSS: when considering two (or more) secrets, the sum of the shares represents a share of the sum of the secrets. In PP-Overgrid, we have applied SSS (and its well-known privacy properties) to the Overgrid scenario to protect user’s consumption data.

For each sub-node, random values of power demand are extracted from uniform distribution in the range $\left[0,p_{max}\right]$, where $p_{max}$ is the maximum power of the nodes, while negative values take into account the presence of power generators. Thus, to retrieve the real consumption data of node *i*, it is necessary to know all the power demands of its sub-nodes. This can be easily avoided by opportunistically building the topology connecting sub-nodes to external nodes, i.e., by avoiding all the internal sub-nodes from being connected to the same external node. By executing the Flow Updating schemes between N·n virtual nodes of the Overgrid, the average power demand of the whole Overgrid is equal to the value p/n, from which *p* can be derived.

### 4.1. Architecture Extension

**Figure 4:**
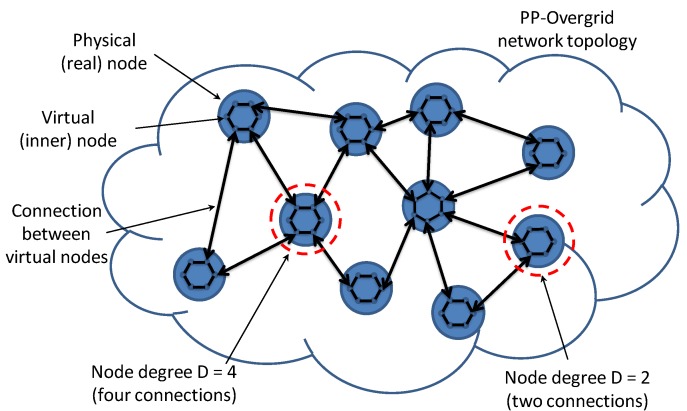
PP-Overgrid logical topology showing the internal ring within the physical node.

The idea of using virtual nodes inside each physical node belonging to the Overgrid allows for easily extending the distributed DR scheme, without changing the messages and the steps of the scheme. The only difference from the original version is related to the execution of additional computations at each node, related to the presence of the internal sub-nodes. This additional complexity is negligible, with each step of the flow updating algorithm being based on simple sums. Moreover, the number of real messages transmitted over the network does not change if we build a topology in which, at each node *i*, only one sub-node is connected to each neighbor node, as shown in Figure 4.

Similarly to the topology considered in the Multi-party algorithm presented in [29,30], sub-nodes are sequentially connected in a ring topology inside each real node. This design choice makes the internal steps easy to be implemented and guarantees that the overlay network is not partitioned. Different design choices avoiding network partitions can also be considered.

Obviously, if a node is isolated from the rest of the network, the Flow Updating scheme executed by the sub-nodes on the internal ring converges to the actual consumption value of the physical node. Conversely, if the sub-nodes are connected with other sub-nodes of neighboring nodes, then the algorithm mixes such information with the consumption of the other nodes, averaging the shares of the nodes with the shares of the neighbors. Thus, in order to preserve the users’s privacy, in PP-Overgrid, the internal execution of the Flow Updating algorithm on virtual sub-nodes is triggered only after the reception of messages by all the neighboring nodes. This guarantees that, even if a tagged node is isolated for a while, the execution of internal averaging operations is suspended too and will not reveal the power demand of the node one the external connection is re-established.

It is easy to see that the privacy level of the proposed scheme depends on the number of node’s neighbors, also called node degree. Indeed, sub-nodes communicate some of the power demand shares to other neighboring nodes. If all of the neighboring nodes of a target node collude with each other, i.e., exchange the messages received by the sub-nodes belonging to the target node, then they can retrieve the secret power demand of the node. In more detail, the SSC scheme guarantees that no information is disclosed until the shares of the sub-nodes are kept separated (i.e., not summed together). To converge, the Flow Updating algorithm indirectly "mixes" and sums the shares of all the sub-nodes in the network, revealing the average value of all nodes together. However, the consumption of a target node can still be revealed if *all* its neighbors collude to compute the sum of the flows towards the target node. Indeed, if the Flow Updating has converged, the colluding node’s estimate will be the same of the target node’s estimate which, according to Equation 1, is equal to the node’s (secret) value minus the sum of the flows. Otherwise, no information is revealed, as explained in [29].

### 4.2. Convergence Time

**Figure 5:**
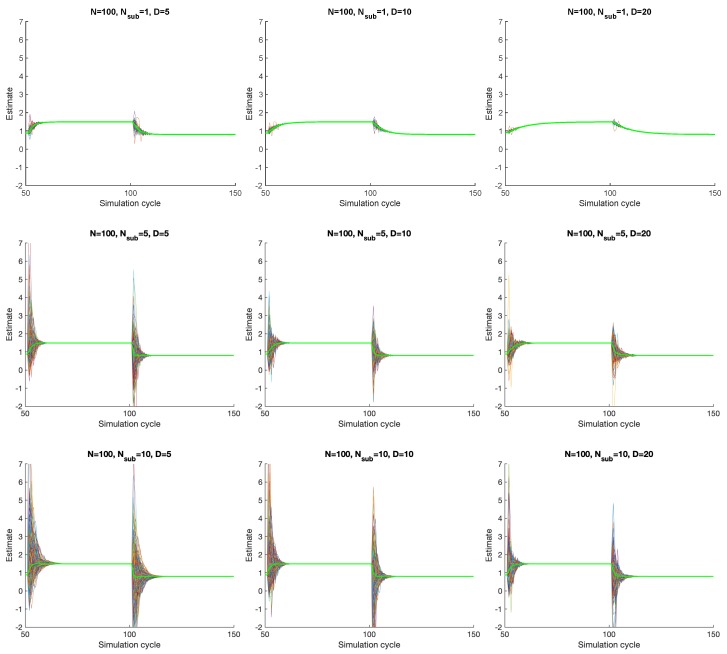
Convergence of the PP-Overgrid algorithm with N=100 nodes, subnodes n={1,5,10} and graph degree D={5,10,20}.

Thanks to the scalability of Overgrid, creating virtual subnodes does not increase the convergence time. Instead, it slightly improves it, although it also increases the variability of intermediate results before convergence. To verify this, we simulate PP-Overgrid on a network of 100 nodes without sub-nodes (n=1, i.e., no privacy) and with 5 or 10 sub-nodes in each node. The topology of the network is a random graph, with a fixed node degree *D* between 5 and 20, i.e., *D* is the minimum number of connections per node. In this experiment, we assume that all nodes exchange messages and execute a step of the Flow Updating scheme at regular time intervals, called cycles. Figure 5 shows the average power consumption *p* estimated over time for all the nodes of the grid, as well as the global network average (think green line). In the experiment, we start from a stationary state, and then the power demand of each node is suddenly changed to a new value at simulation cycles 50 and 100.

From the figure, it is clear that, without sub-nodes (top three figures), the convergence of PP-Overgrid is slower while, with a higher number of sub-nodes, the convergence is faster. The reason relies on the fact that increasing the number of sub-nodes significantly reduces the degree of the PP-Overgrid network (the external connections are distributed among the different sub-nodes). Indeed, as shown in [15], the convergence time of the Flow Updating algorithm will improve if the network is loosely connected (and this is what happens when creating the sub-nodes). Additionally, note that the global average (thick green line) converges quicker with more sub-nodes, but, taking a closer look, the overall network convergence speed is slower: indeed, the single nodes (local average) need more iterations to get closer to the global network average. Nevertheless, even in the presence of 10 sub-nodes (i.e., increasing the number of nodes by an order of magnitude), the increase in the convergence time is negligible, proving the feasibility of PP-Overgrid.

## 5. Experiments

**Figure 6:**
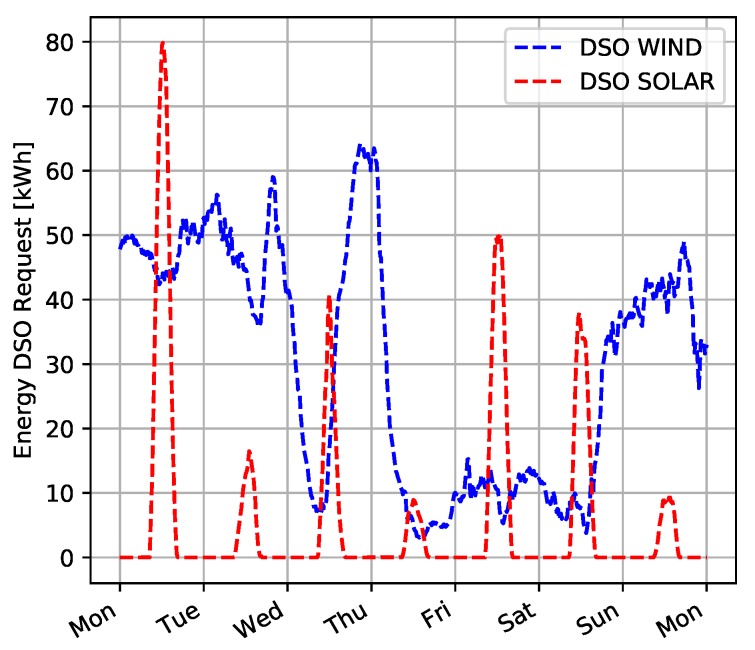
Total wind and solar energy production during a week.

A possible application of PP-Overgrid is to compensate the variability of energy production from unpredictable sources, such as wind and solar. For example, Figure 6 shows the total wind and solar energy production in Belgium, obtained by analyzing publicly available data of the Belgium’s transmission system operator [31]. From the figure, it is clear that such energy sources, and particularly wind, are highly variable and difficult to predict [32]. In our experiments, we will derive the power constraint $P_{DSO}$ applied by the operator by considering these data about renewable energy production.

PP-Overgrid has been implemented in Java exploiting the PeerSim simulator [33], which provides the basic tools to simulate the gossip P2P overlay. The topology is generated randomly by PeerSim, where the number of nodes represent the number of smart buildings, while, for the power consumption data, we used the traces described in Section 5.2. Running independently on each node, the gossiping protocol exchanges messages with the neighbor nodes, transmitting the information required for the Flow Updating algorithm. At startup, the emulator generates the random topology of the P2P network, with average node degree D the number of neighbors per node. It builds the connections between the nodes (each node has a neighbor “list”), so that each node then communicates to the neighboring nodes based on this topology. The list of neighbors can be statically configured or can change dynamically as nodes join and leave the overlay. For easiness of presentation, however, in our experiments, we limit our analysis to a static environment.

### 5.1. Characterizing Flexible Loads

For the implementation of the DR programs, it is necessary to distinguish loads that are flexible and, therefore, manageable, from those that are not flexible. In residential environments, loads that may be considered flexible include:

- Heating and cooling units
- Washing machines
- Dryers
- Dishwashers
- Water heaters

while in the category of non flexible loads fall:

- Lights
- Air circulation pumps for AC
- Televisions and decoders
- Computers
- Fridges and freezers

The management of flexible loads such as washing machines and dishwashers is easily achievable (e.g., through smart plugs). The controlled interruption of the air conditioning units for short time does not have a particularly negative influence on perceived comfort conditions: switching off the heating and/or cooling for small periods does not produce an abrupt change in temperature in the rooms, but a gradual change which can be corrected as soon as the air conditioning system is reactivated. In residential buildings, there is a multiplicity of other electrical loads that are, however, discontinuous and are active for short intervals of time; their extreme variability makes them impossible to be included in the list of flexible loads. For other commercial buildings, the loads that may be considered flexible are only the AC units.

The traces described in Section 5.2 have been divided between flexible and non-flexible load shares. The construction of such load profiles was carried out considering the average power absorbed by flexible (FLEX) loads and for non flexible loads (NOFLEX). Knowing the FLEX/NOFLEX load diagrams, it is then possible to use the traces to test PP-Overgrid applying the DR program to the flexible power only.

Energy consumption often exhibits regular patterns, with similar daily and weekly patterns. In our experiments, we assumed that every 15 min the DSO checks the energy production and requests from the PP-Overgrid users a modification (up or down) in the global power consumption of the grid. We then simulate the behavior of the nodes (each with a different amount of flexible power) responding to the power change requests made by the DSO.

### 5.2. Energy Load Profiles

We use real energy production traces from renewable sources, obtained by analyzing the wind/solar generation data in Belgium [31]. For the smart buildings, we also use real world power consumption traces and explicitly distinguish the flexible and non-flexible appliances. In particular, for the power consumption traces, we used NEEA’s RBSA dataset [34], a comprehensive metering study covering most energy end uses in over 100 residential homes. The target loads included in the RBSA metering are:

- Heating and cooling
- Hot water
- White goods/appliance: refrigerator, freezer, clothes washer, clothes dryer
- Consumer electronics: TV, TV accessories and Computer, computer accessories
- Lighting
- Other large loads: hot tubs, well pumps, sump pumps, electric cars, etc.

**Figure 7:**
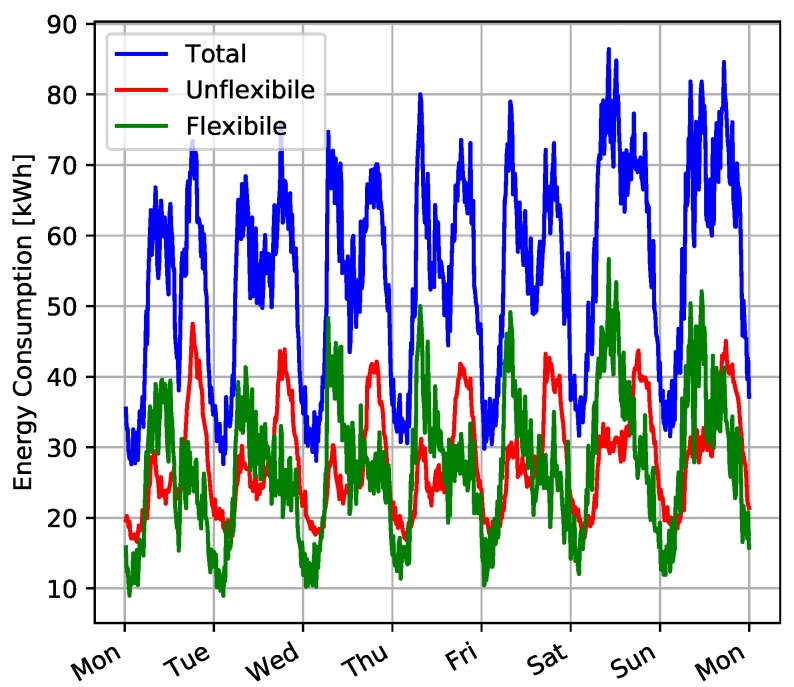
Total, flexible, and inflexible power consumption during a sample week in the NEEA’s RBSA dataset.

We analyzed these consumption traces to distinguish the flexible power from non-flexible power. For example, Figure 7 shows the total, flexible, and non-flexible power consumption reported in the RBSA dataset for a sample week.

The detailed list of loads considered as flexible is detailed in Table 1 and considers all the three housing types of the RBSA dataset: single-family homes, manufactured homes, and multifamily buildings. The table captures data mostly from Heating, Ventilation and Air Conditioning (HVAC) systems but also appliances such as washing machines, dryers, dishwashers, and DHW (Domestic Hot Water) systems. The use of such data allows us to validate the feasibility of PP-Overgrid in realistic scenarios, monitoring the performance of the P2P network, and analyzing the nodes consumption with and without applying the DR program.

### 5.3. Numerical Results

In this section, we analyze the ability of PP-Overgrid to respond to realistic DSO requests, by considering 10 sub-nodes for each real node joining the Overgrid, a total number of N=100 real nodes and a network degree of 5 or 20. We assume that the DSO updates its power constraint signal every 15 minutes and that each node can adapt the consumption of flexible loads in a purely proportional fashion. In other words, $p_{i}$ being the initial power demand of node *i* and $p_{i\_new}$ the power demand after the DR scheme, we consider that it is feasible to perform a proportional reduction $p_{i_{new}}=\frac{P_{DSO}}{pN}\cdot p_{i}$ at each node *i*, in order to meet the DSO request $P_{DSO}$.

**Figure 8:**
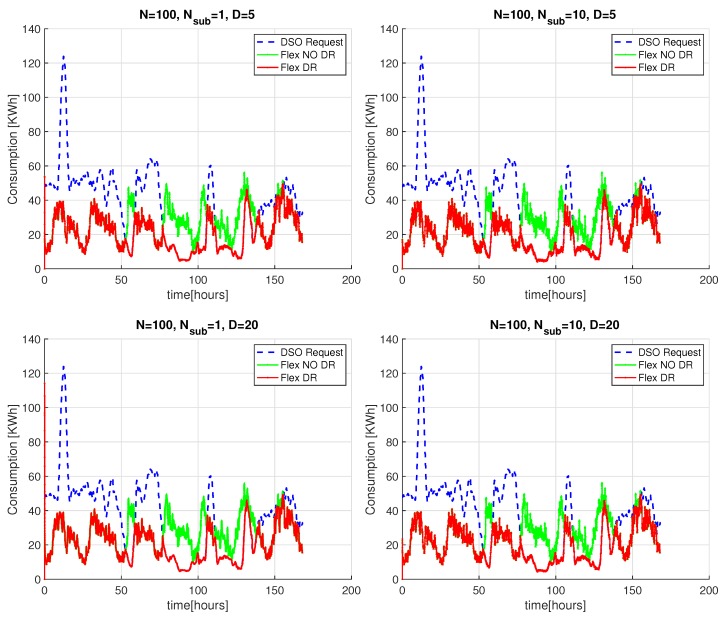
DSO adaptation of PP-Overgrid algorithm with N=100 nodes, subnodes n={1,10} and graph degree D={5,20}.

**Figure 9:**
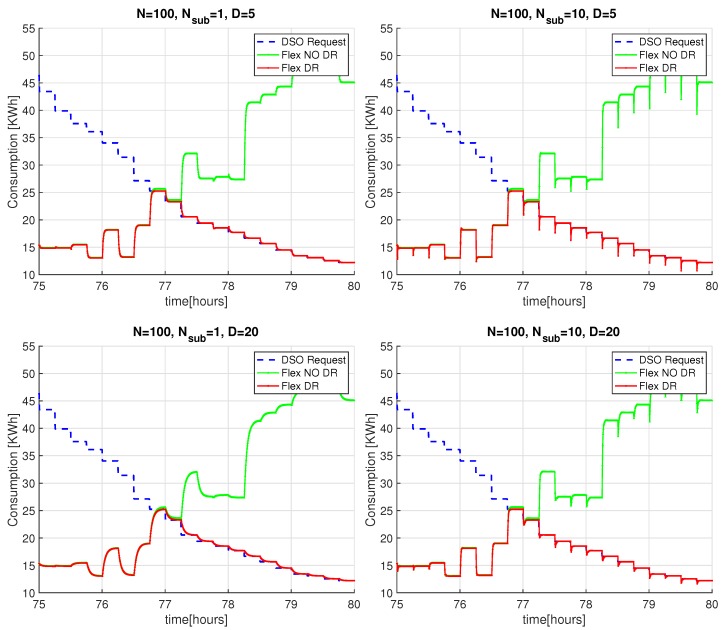
Detail of the PP-Overgrid convergence adaptation with N=100 nodes, subnodes n={1,10} and graph degree D={5,20}.

Figure 8 shows the aggregated power demand of PP-Overgrid when applying the DR (red curve) and without applying the DR scheme (green curve), together with the $P_{DSO}$ signal (blue curve) sent by the DSO. For completeness, the figure also shows the results without applying the privacy preserving scheme, i.e., without sub-nodes (n=1). In all the considered scenarios, for a network degree D=5 or D=20, the results show that the DR scheme is able to meet the DSO constraints, since the red curve closely matches the blue one. This fact is better depicted in Figure 9, where we only show results for the time interval between the 75th and the 80th hour, improving the readability of the comparison between the blue and red curves. In such a figure, we can also appreciate the convergence time of the scheme for network topologies of different degrees *D*: as anticipated in Section 4.2, the lower the degree of the network, the faster the computation of the average power demand (and consequently, the faster the computation of the adaptation actions). Note also that, for D=20, the introduction of the internal sub-nodes improves the convergence of the scheme because it reduces the degree of the overall topology, including both virtual and real nodes.

### 5.4. Quantized Load Adaptation

The proportional control of the loads might not always be feasible in practical cases because appliances often can only be switched on or off. To overcome this problem, we propose an alternative control scheme, based on *statistical* decisions about the activation/de-activation of the loads. To meet the DSO constraint, in case the power constraint $P_{DSO}$ is higher than the estimated power demand P=p·N·n of the Overgrid, users could randomly switch off each appliance with probability:

(2) $$Pr\left\{off\right\}=1-\frac{P_{DSO}}{p\cdot n\cdot N}.$$

**Figure 10:**
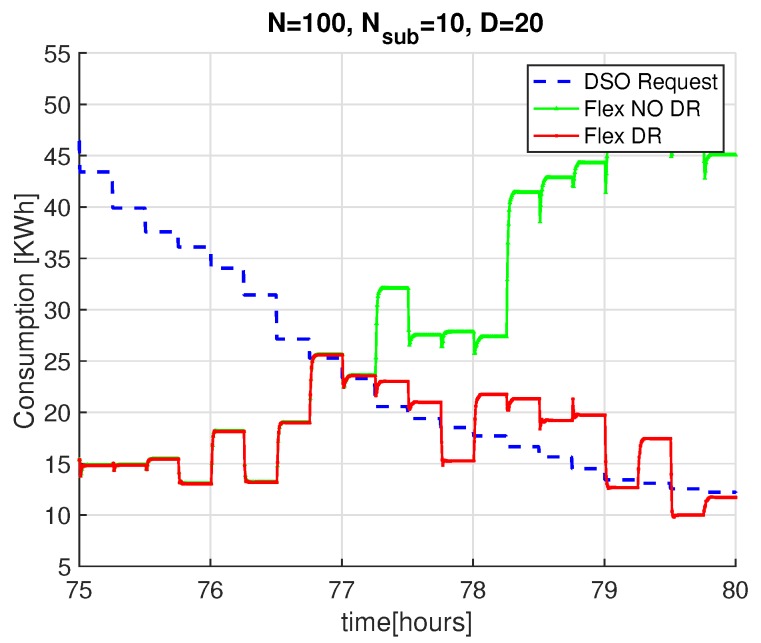
Detailed results of the quantized DSO Adaptation, with subnodes n=10 and graph degree D=20.

**Figure 11:**
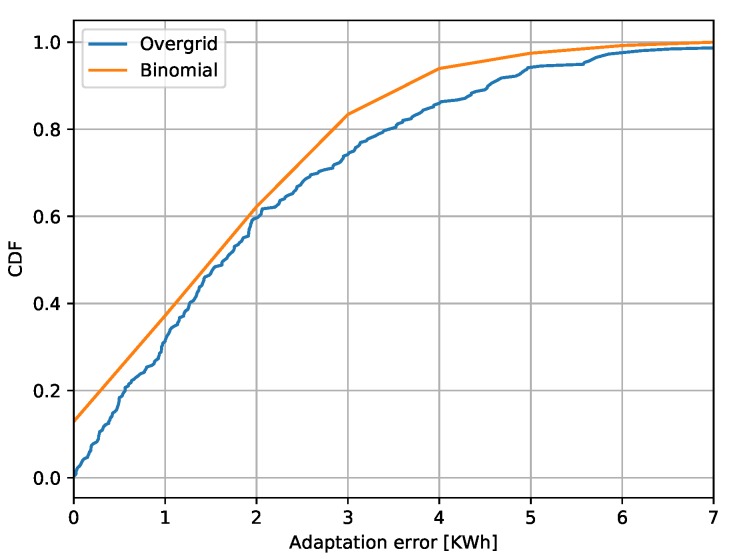
CDF of the DSO adaptation errors caused by quantized loads and comparison with the binomial distribution.

If the number of nodes (and appliances) is large enough, the effect of such random decisions will make the total power demand close to $P_{DSO}$. Figure 10 shows again the DSO constraint over time (blue curve), together with the total power demand of a network with N=100 nodes and D=20, with (red curve) or without (green curve) activating the DR scheme based on this statistical load control. The figure shows that the adaptation (red curve) closely follows the DSO requests variation, although the two curves do not perfectly match because of the random decisions and discrete values of the consumption loads. To quantify the mismatch between the desired $P_{DSO}$ power demand and the one achieved by PP-Overgrid, Figure 11 shows the Cumulative Distributed Function (CDF) of the absolute error $|P-P_{DSO}|$. From the figure, it is evident that the adaptation error is lower than 5 kWh in more than 90% of the cases. The figure also shows the CDF of the corresponding Binomial function, showing that the errors follow the random process of touring the loads off, with approximately the same distribution. This means that the error can be controlled and minimized by increasing the number of nodes in the network, as well as reducing the size (quantization) of the loads.

## 6. Conclusions

In this paper, we proposed PP-Overgrid, a scalable solution for supporting privacy-preserving DR schemes working in a community of smart buildings, acting as a virtual microgrid regardless of their physical location. Based on a distributed P2P protocol, called Flow Updating, PP-Overgrid is able to estimate of the total power demand of the grid without violating the single users’ power profiles. To compute such a power demand in a privacy-preserving manner, we introduced virtual sub-nodes inside each real node of the original Overgrid. Similar to Secure secret sharing algorithms, sub-nodes communicate to the neighboring nodes randomly generated consumption data, guaranteeing that the total sum of aggregated data is equal to the real one.

Additionally, PP-Overgrid implements two different types of distributed load control to adapt the power consumption of the network to the power constraints communicated by the DSO: a proportional (continuous) reduction of the load and a quantized (probabilistic) activation/de-activation of the loads. We explored the applicability of our scheme using a P2P network simulator and real power consumption traces of residential buildings. Our results show the feasibility of the scheme in matching a desired power constraint, under different network topologies and scenarios.

## Figures and Tables

**Table 1:** Description of residential loads identified as flexible.

#	Name	Type	Description
1	AC	HVAC	Central air conditioner outdoor unit energy use in kWh
2	Boilr_g_e	HVAC	Hydronic loop electric pump energy use in kWh
3	DHP	HVAC	Ductless heat pump total energy use in kWh. Includes both outdoor and indoor units
4	ER	HVAC	Zonal electric resistance heater energy use in kWh.
5	ER_2	HVAC	Additional zonal electric resistance heater energy use in kWh.
6	ER_3	HVAC	Additional zonal electric resistance heater energy use in kWh.
7	ER_4	HVAC	Additional zonal electric resistance heater energy use in kWh.
8	ER_5	HVAC	Additional zonal electric resistance heater energy use in kWh.
9	Furn	HVAC	Electric furnace resistance heating element energy use in kWh
10	Furn_AH	HVAC	Central forced air system air handler energy use in kWh. Includes air handlers for gas furnaces, electric resistance furnaces, central air conditioners, and heat pumps.
11	HP_in	HVAC	Air source heat pump system auxiliary resistance element energy use in kWh. Located indoors; at the air handler, the elements provide additional heat.
12	HP_in_2	HVAC	Additional air source heat pump system auxiliary resistance element energy use in kWh. Located indoors, at the air handler, the elements provide additional heat
13	HP_out	HVAC	Air source heat pump outdoor unit energy use in kWh. Records energy use for both heating and cooling.
14	HP_out_2	HVAC	Additional air source heat pump outdoor unit energy use in kWh. Records energy use for both heating and cooling.
15	PTAC	HVAC	Packaged terminal air conditioner energy use in kWh
16	PTHP	HVAC	Packaged terminal heat pump energy use in kWh
17	Dryer	Appliance	Clothes dryer energy use in kWh. Includes heating element, drum motor, and exhaust fan energy.
18	Dwash	Appliance	Dishwasher energy use in kWh.
19	Cwash	Appliance	Clothes washer energy use in kWh
20	Cwash_2	Appliance	Additional clothes washer energy use in kWh
21	DHW_1	DHW	Primary electric resistance tank water heater energy in kWh
22	DHW_2	DHW	Secondary electric resistance tank water heater energy in kWh
23	DHW_HP	DHW	Heat pump water heater energy use in kWh.
